# Genome-Wide Analysis of *SRNF* Genes in *Gossypium hirsutum* Reveals the Role of *GhSRNF18* in Primary Root Growth

**DOI:** 10.3389/fpls.2021.731834

**Published:** 2021-09-22

**Authors:** Li Yu, Shuojun Zhang, Hailun Liu, Yufei Wang, Yiting Wei, Xujiao Ren, Qian Zhang, Junkang Rong, Chendong Sun

**Affiliations:** ^1^The Key Laboratory for Quality Improvement of Agricultural Products of Zhejiang Province, College of Advanced Agricultural Sciences, Zhejiang Agriculture and Forestry University, Hangzhou, China; ^2^The State Key Laboratory of Subtropical Silviculture, College of Forest and Biotechnology, Zhejiang Agriculture and Forestry University, Hangzhou, China

**Keywords:** genome-wide characterization, *SRNF* gene family, *Gossypium hirsutum*, PR growth, RAM activity

## Abstract

Root systems are instrumental for water and nutrient uptake and the anchorage of plants in the soil. Root regulating GL2-interacting repressors (GIRs) contain a Short RING-like Zinc-Finger (SRNF) domain, but there has been no comprehensive characterization about this gene family in any plant species. Here, we renamed the GIR-like proteins as SRNF proteins due to their conserved domain and identified 140 *SRNF* genes from 16 plant species including 24 *GhSRNF* genes in *Gossypium hirsutum*. Phylogenetic analysis of the SRNFs revealed both similarities and divergences between five subfamilies. Notably, synteny analysis revealed that polyploidization and whole-genome duplication contribute to the expansion of the *GhSRNF* gene family. Various cis-acting regulatory elements were shown to be pertinent to light, phytohormone, defense responsive, and meristem regulation. Furthermore, *GhSRNF2*/*15* were predominantly expressed in root, whereas the expression of *GhSRNF18* is positively correlated with the primary root (PR) length in *G. hirsutum*, quantified by quantitative real-time PCR (qRT-PCR). Over-expression of *GhSRNF18* in *Arabidopsis* and virus-induced gene silencing (VIGS) of *GhSRNF18* in *G. hirsutum* has revealed the role of GhSRNF18 in PR growth. The over-expression of *GhSRNF18* in *Arabidopsis* resulted in an increase of meristematic activities and auxin accumulations in PRs, which were consistent with the transcriptomic data. Our results suggested that *GhSRNF18* positively regulates PR growth. This study increased our understanding of the *SRNF* gene family in plants and provided a novel rationale for the further investigation of cotton root morphogenesis regulated by the *GhSRNFs*.

## Introduction

Roots are vital organs in terrestrial higher plants, not only for water and nutrient uptake and for the anchorage of plants in the soil, but to protect plants from diverse pathogens (Petricka et al., 2012; Rich-Griffin et al., 2020). Plant roots are underground organs developed from the root apical meristem initiated during embryogenesis (Motte et al., 2019). Root systems consist of two principal root types, namely, the primary root (PR), which is formed embryonically, and secondary roots (SRs), which form post-embryonically (Olatunji et al., 2017). Total root growth depends on two processes: cell elongation in the elongation zone and cell proliferation in proximal meristems (Camacho-Cristobal et al., 2015). The quiescent center (QC), a group of cells that divide infrequently and from which all tissue systems of the root originate, is required to maintain asymmetric divisions of surrounding stem cells and prevent their differentiations non-cell-autonomously signaling (Kidner et al., 2000; Ten Hove and Heidstra, 2008). The *Wuschel-Like Homeobox 5* (*WOX5*) gene is expressed mainly in the QC to prevent QC cell proliferation by repressing the expression of *CYCD3;3* (Motte et al., 2019). From meristematic zone (MZ) to transition zone (TZ), the activity of cell division decreased gradually with the increase of distance from the stem cell niche (Petricka et al., 2012). Therefore, the position of TZ determines the size of the meristem, which is directly related to the rate of root growth (Petricka et al., 2012). The *HOBBIT*/*Cell Division Cycle 27 Homolog B* (*CDC27B*) encodes a core component of the APC/C complex and plays a crucial role in mitotic activity maintenance of root apical meristem (RAM) (Blilou et al., 2002; Serralbo et al., 2006; Perez-Perez et al., 2008). The PLETHORA (PLT) and SHORTROOT (SHR)/SCARECROW (SCR), which represented the AP2 and GRAS transcription factors, respectively, are involved in the establishment of the QC identity and root stem cell activities by two parallel pathways (Petricka et al., 2012). In *Arabidopsis, scr* and *shr* loss-of-function mutants exhibit a shorter primary root and loss of QC identity (Helariutta et al., 2000; Nakajima et al., 2001). Meanwhile, *PLT1* and *PLT2* in *Arabidopsis* are redundantly required for distal cell division patterns and stem cell maintenance in root meristem in an auxin-dependent manner (Aida et al., 2004). Rapid cell expansion in the elongation zone (EZ) contributes to primary root growth and is largely regulated by plant hormones, including abscisic acid (ABA), brassinosteroids (BR), cytokinin, ethylene, gibberellic acid (GA), and auxin (IAA) (Petricka et al., 2012). The rapid expansion of cells in EZ involves the process of vacuole swelling and cell wall remodeling (Schiefelbein and Somerville, 1990; Ludevid et al., 1992; Arioli et al., 1998; Schumacher et al., 1999; Cosgrove, 2000; Darley et al., 2001; Bennett et al., 2010). In elongation zone cells, vacuole swelling depends on the tonoplast aquaporin Gamma Tonoplast Intrinsic Protein (GAMMA-TIP), which is specifically expressed in the EZ (Ludevid et al., 1992). Loss of function mutations in cellulose synthesis, such as *rsw1*-*3* and procuste, result in cell expansion defects (Arioli et al., 1998; Fagard et al., 2000).

In *Arabidopsis*, GL2-interacting repressor 1 (GIR1) and GL2-interacting repressor 2 (GIR2) have been identified recently as novel plant-specific proteins, which negatively regulate the root hair development through interaction with GLABRA2 (GL2) (Wu and Citovsky, 2017a). In *Gossypium arboreum, GaFzl*, a gene homologous to the *GIR1* in *Arabidopsis*, has been proved to be closely associated with trichome and fuzz development (Feng et al., 2019; Wang et al., 2021). However, the GIR family proteins have not been defined. Besides, the conserved domain and biological function of GIR family members remain unclear. In this study, we systematically identified 140 *GIR-like* genes in dicotyledons, monocotyledons, moss, and ferns, all of which share a conserved Short RING-like Zinc-Finger domain (CX_2_CX_12_CX_2_X_10_). As such, we redefined GIR-like protein as Short RING-like Zinc-Finger domain contained (SRNF) protein. Then, we systematically identified 24 *SRNF* family members in *Gossypium hirsutum* and analyzed their phylogenetic relationships, protein structure, chromosomal locations, conserved motif distribution patterns, cis-acting elements, gene collinearity, Ka/Ks values, and expression patterns. Moreover, through association analysis, we found a positive correlation between the *GhSRNF18* expression level and the PR growth in several upland cotton cultivars. Besides, overexpression of *GhSRNF18* in *Arabidopsis* resulted in an increase of PR length and *GhSRNF18* VIGS exhibited the opposite phenotype, which further confirmed the role of GhSRNF18 in PR growth. Finally, we found that GhSRNF18 modulates the PR growth by regulation of root meristematic activities and endogenous auxin levels in PRs.

## Materials and Methods

### Plant Materials and Treatment

Wild-type *Arabidopsis thaliana Columbia-0* (*Col-0*) plants were used to generate transgenic *OE-GhSRNF18* lines. Plants were screened on 1/2 Murashige and Skoog (MS) medium under long-day conditions (16-h light/8-h dark cycle) at 23°C. Root phenotypes were measured on the 1/2 MS medium for 20 replicates until 14 d. Upland cotton (*G. hirsutum acc*. *TM*-*1, HM1, NLD402, NLD18, NLD19, NLD20*) seeds were surface sterilized with 70% (v/v) ethanol for 1 min and 10% hydrogen peroxide for 2 h, followed by washing with sterile water. For subsequent qRT-PCR, cotton seeds were germinated on the 1/2 Murashige and Skoog (MS) medium, then transplanted into the sand culture (vermiculite) under 15,000 Lux light (12-h light/12-h dark cycle) at 23°C.

### Identification and Property Analysis of *GhSRNF* Genes

The GL2-interacting repressors protein sequences in *Arabidopsis* were obtained from TAIR 10^1^. The retrieved AtGIR protein sequences were then used as queries to identify orthologous genes in *G*. *hirsutum* (ZJU, version 2.1), *Gossypium arboreum* (WHU, version 3.0), *Gossypium raimondii* (NSF, version 1.0). All cotton genome databases were downloaded from CottonGen^2^ (Yu et al., 2014). Then, the conserved amino acid residues were explored by SMART^3^ (Letunic et al., 2015). Basic properties of *GhSRNF* proteins were estimated using ExPASy Compute pI/Mw tool^4^. The subcellular localization was predicted by Plant-Ploc^5^ (Chou and Shen, 2008). Further, other plant genome databases used in this study were downloaded from Phytozome v12.1^6^.

### Multiple Alignments and Phylogenetic Analysis

Multiple alignments of amino acid sequences were performed by MUSCLE with default parameters. For phylogenetic analysis, two phylogenetic trees were constructed using MEGA X (version 1.01, PSU, PA, USA) with Neighbor-Joining (NJ) methods (Kumar et al., 2018). Moreover, 1,000 bootstrap replicates were used to determine support values for the inferred phylogenetic trees. Phylogenetic trees were then visualized by EvolView v3^7^ (Subramanian et al., 2019).

### Conserved Motif Distribution, Cis-Acting Elements Analysis

The software MEME v5.3.3^8^ (Bailey et al., 2009) was used for conserved motifs identification. The upstream 2.0 kb DNA sequence of the *SRNF* genes in cotton was extracted to predict cis-acting regulatory elements on PlantCARE^9^. Conserved motifs distributions were drawn by Tbtools Gene Structure View (Chen et al., 2020).

### Chromosomal Mapping and Synteny Analysis

Chromosomal position information for all *GhSRNFs* was obtained from annotation files downloaded from the CottonGen (see footnote 2) website (Yu et al., 2014). The MCScanX software (version Nov. 11, 2013, Athens, GA, USA) was used to analyze SRNF genes synteny and collinearity relationships between *G. hirsutum*, *G. arboreum*, and *G. raimondii* (Wang et al., 2012). Chromosomal gene mapping and Circos figure described previously were visualized by Tbtools. Also, Ka/Ks values were calculated by Tbtools Ka/Ks Calculator (Chen et al., 2020).

### *GhSRNF* Gene Expression Patterns Analysis

The *GhSRNF* gene expression datasets were acquired from public high-throughput datasets in Cotton Omics^10^, comprising 12 different tissues (root, stem, leaf, bract, sepal, torus, filament, anther, pistil, ovule, fiber) at different developmental stages in *G. hirsutum acc*. *TM*-*1* (Hu et al., 2019). The read counts had already been generated, FPKM (FPKM, Fragments Per Kilobase of exon per Million mapped reads) values were calculated for the quantification of gene expressions. The clustered heatmap was visualized with FPKM (log_2_) of *GhSRNF* genes by Tbtools using Heatmap illustrator (Chen et al., 2020).

### RNA Isolation and qRT-PCR

Total RNA was extracted from 0.10 g of cotton PR samples using RNAprep pure Plant Kit (code: DP432, Tiangen, Beijing, China) based on the instructions of the manufacturer. Reverse transcription was performed using Hifair 1st Strand cDNA Synthesis SuperMix for qPCR (YEASEN Biotech, Shanghai, China) with the same amount of total RNA (1μg). The gDNA digester mix was used to remove gDNA contamination. The qRT-PCR was performed using Hieff qPCR SYBR GreenMaster Mix (YEASEN Biotech, Shanghai, China). The reaction parameters were as follows: 95°C for 5 min, followed by 40 cycles of 95°C for 10 s and 60°C for 30 sec. The cotton ubiquitin gene *UBQ7* was used as the internal control for each sample. The relative expression levels were calculated using the 2^−ΔΔCt^ method. Primers for qRT-PCR are listed in Supplementary Table 1, and one-way ANOVA statistical analysis was then performed.

### Binary Vector Construction, Transgenic Lines Generation

For ectopic expression of *GhSRNF18*, the open reading frame (ORF) of *GhSRNF18* was amplified using the gene-specific primers listed in Supplementary Table 1 and cloned into the *pCAMBIA1300* vector (CAMBIA). The binary construct was transformed into *Agrobacterium tumefaciens* strain GV3101. *Arabidopsis* transformation was carried out using the floral dip method. Transgenic *Arabidopsis* seeds were screened on 1/2 Murashige and Skoog (MS) medium suspended with hygromycin (30 mg/L).

### VIGS Assay

For VIGS, full-length ORF of *GhSRNF18* (255 bp) was cloned into *pTRV2* (tobacco rattle virus), thus constructing *pTRV2*: *GhSRNF18* vectors. Primers for construction and identification were listed in Supplementary Table 1. VIGS injection was displayed until cotyledons fully expanded, infection solution containing *Agrobacterium tumefaciens* of *pTVR1* and *pTRV2*: *GhCLA* (positive control) or *pTRV1* and *pTRV2*: *GhSRNF18* or *pTRV1* and *pTRV2* (negative control). Plant materials were cultured under the condition above until positive control exhibited the albino phenotype.

### Protoplast Subcellular Localization

The *Arabidopsis* protoplast isolation protocol and transfection methods were performed as described before (Yoo et al., 2007). GhSRNF18 fused GFP (green fluorescent protein) with NLS-mCherry (nuclear localization sequence-fused protein mCherry) were co-transformed into *Arabidopsis* protoplast. The GFP and mCherry in protoplast were photographed using a fluorescence microscope Imager A2 (Zeiss, Oberkochen, Germany).

### RNA-seq, KEGG, and GO Analysis

Total RNA was isolated from root tissues of *OE-GhSRNF18*/*Col-0* lines and WT/*Col-0* grown for 20 days after germination. The fragments were purified by agarose gel electrophoresis and sequenced with NovaSeq 6000 Sequencer (Illumina Inc., San Diego, CA, USA) with a read length of 150 bp. Three biological replicates were performed separately. We used Hisat2 to align the reads to TAIR 10 genome annotation. Gene read counts were assembled by StringTie with the union mode, and differential expression genes (DEGs) analysis was performed by DEseq2, FPKM > 1.0 (FPKM, Fragments Per Kilobase of exon per Million mapped reads) were regarded as valid DEGs. The up-regulated and down-regulated genes were used for KEGG and GO enrichment separately by OmicShare tools^11^. The raw transcriptome sequencing data were available in the NCBI SRA (Sequence Read Archive) under the BioProject accession ID PRJNA743223.

### Determination of IAA Contents

*Arabidopsis* root samples harvested at 2 weeks were frozen and ground into powder immediately. The mass fractions of indole-3-acetic acid (IAA) were determined using an enzyme-linked immunosorbent assay as described before (Shen et al., 2020). Each sample was measured in parallel three times. The standard curve graph was built using IAA concentration contained test samples, the IAA concentration in each gram of sample could be obtained through the antilog.

### EdU Staining

EdU (5-ethynyl-2′-deoxyuridine) was a thymidine analog whose incorporation can be used like BrdU (bromodeoxyuridine) to label cells undergoing DNA replication. *Arabidopsis* root tips were treated for 2 h with 10 μM EdU, fixed, and incubated with EdU detected cocktail. EdU-labeled cells were stained with Click-iT EdU Alexa Fluor Imaging Kit (Invitrogen, Eugene, OR, USA). EdU-labeled nuclei were detected by fluorescence microscope Imager A2 (Zeiss, Oberkochen, Germany) under a green Fluor 488 signal. The vertical distance from the top to bottom EdU-labeled cells was the cell division zone length, which was measured on three parallel lines.

## Results

### Identification of *SRNF* Genes in Three Cotton Species

**Figure 1:**
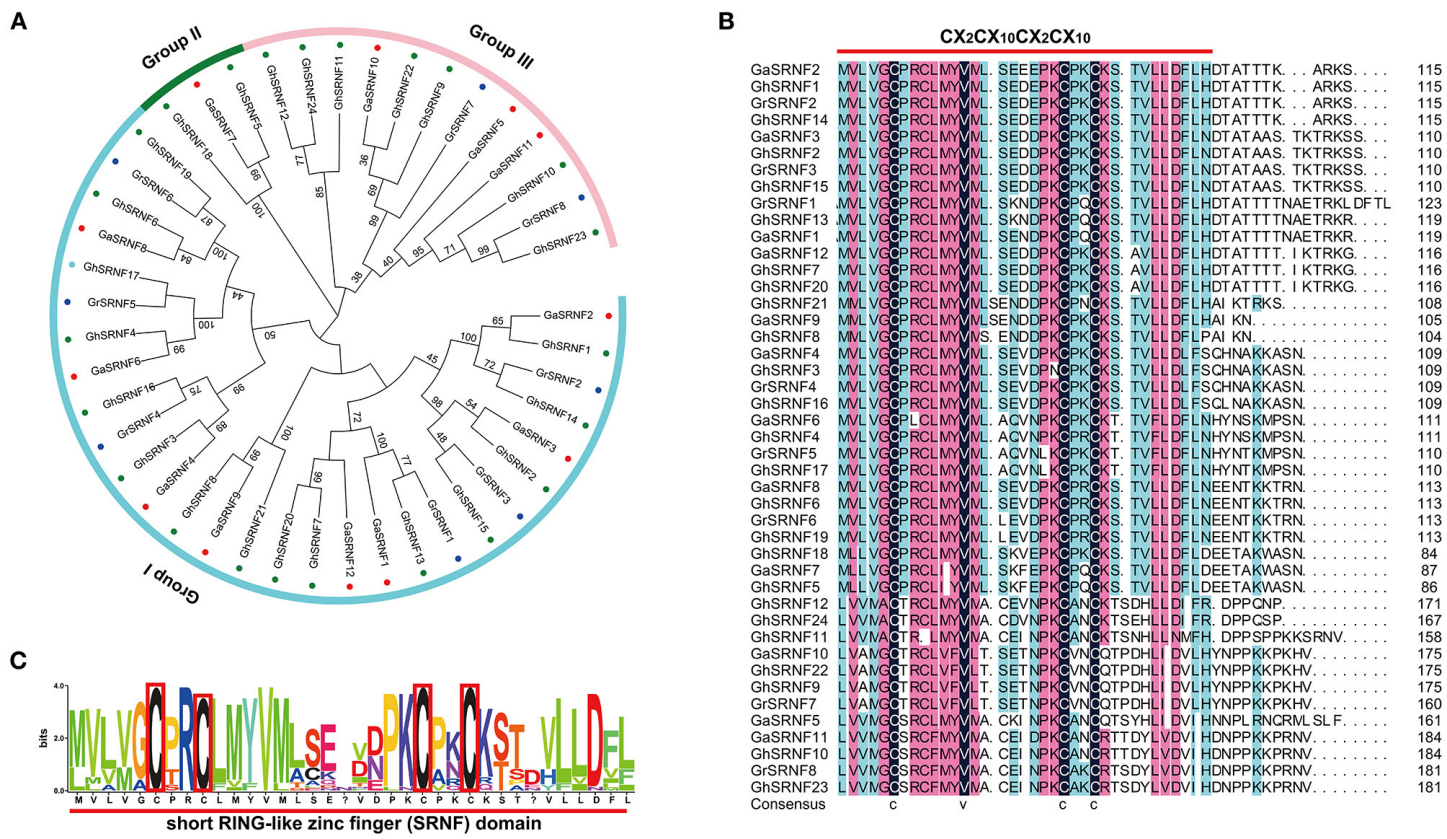
Phylogenetic relationship and domain structure of the SRNF family proteins in three cotton species. **(A)** Neighbor-Joining phylogenetic tree of the SRNF family in *G*. *arboreum, G*. *raimondii*, and *G*. *hirsutum*. **(B)** The conserved Short RING-like Zinc-Finger domain (CX_2_CX_12_CX_2_X_10_). The predicted domain sequence was obtained from CottonGen (see footnote 2). **(C)** Alignment of Short RING-like Zinc-Finger domain (CX_2_CX_12_CX_2_X_10_) in *G. hirsutum*.

To identify all the *GIR* genes in *G*. *arboreum, G*. *raimondii, G*. *hirsutum* genomes, 2 AtGIR protein sequences were employed as queries to perform blast search. A total of 44 *GIR* members were identified in three representative cotton species, of which 12 were *G*. *arboreum* genes, 8 were *G*. *raimondii* genes and 24 were *G*. *hirsutum* genes (Figure 1A). Interestingly, we found that all members of this GIR protein contained a Short RING-like Zinc-Finger domain (CX_2_CX_12_CX_2_X_10_) before the C-terminal (Figures 1B,C). Thus, we renamed them GhSRNF1~GhSRNF24 based on the position of these genes on the chromosome and their conserved domain. According to the unrooted phylogenetic tree in three *Gossypiums, SRNF* genes could be divided into three independent subgroups. *G*. *hirsutum* had more than double the number of *GhSRNF* genes as compared with *G*. *arboreum* and *G*. *raimondii*, illustrating polyploidy and whole-genome duplication (WGD) events during hybridization (Li et al., 2015).

In this study, we mainly focused on the *SRNF* genes in *G*. *hirsutum*. Thus, we collected basic information comprising locus ID, chromosomal position, protein length (aa), molecular weight (MW), isoelectric point (pl), and subcellular localization prediction of SRNF proteins. The length of *GhSRNF* genes encoded proteins ranged from 86 aa (GhSRNF18) to 185 aa (GhSRNF10); the corresponding molecular weight was between 9,345.79 Da to 20,427.61 Da, and pI values varied from 4.34 (GhSRNF5) to 8.95 (GhSRNF14). Moreover, the prediction of subcellular localization and other basic information for all SRNF members in *G*. *arboreum, G*. *raimondii, G*. *hirsutum* were listed in Supplementary Table 2.

### Phylogenetic Relationship Analysis of *SRNF* Genes

**Figure 2:**
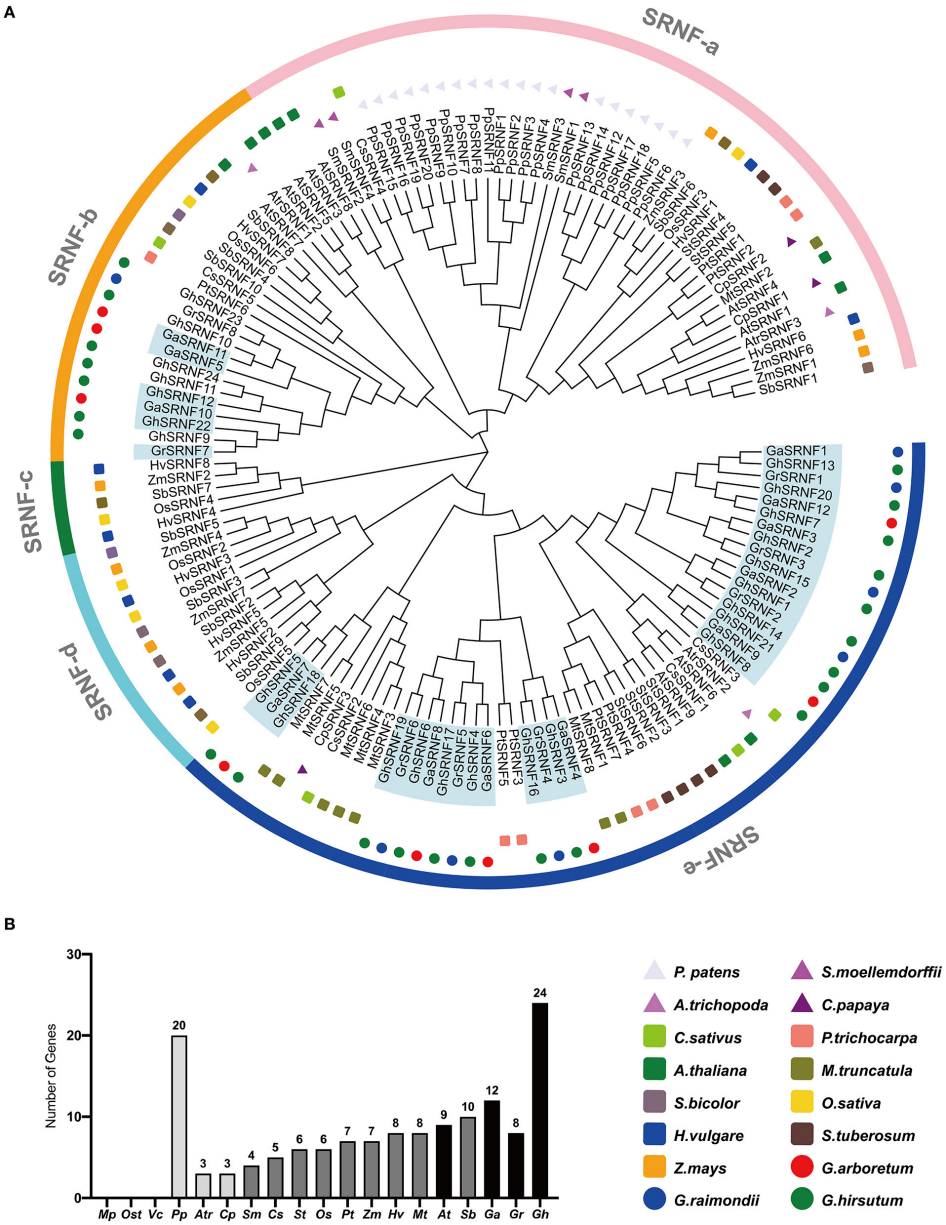
Phylogenetic tree of the *SRNF* family at the amino acid sequence level in 16 plant species. **(A)** The phylogenetic tree resolved all *SRNF* genes from dicotyledons, monocotyledons, moss, and ferns into five major groups from SRNF-a to SRNF-e. **(B)** Comparison of *SRNF* gene numbers across a wide range of plant species above. The prefix *Mp, Ost, Vc, Pp, Atr, Cp, Sm, Cs, St, Os, Pt, Zm, Hv, Mt, At, Sb, Ga, Gr, Gh* were used to describe the names of *M. pusilla, Os. tauri, V. carteri, P. patens, A. trichopoda, C. papaya, S. moellemdorffii, C. sativus, S. tuberosum, O. sativa, P. trichocarpa, Z. mays, H. vulgare, M. truncatula, A. thaliana, S. bicolor, G. arboreum, G. raimondii, G. hirsutum*, respectively.

To excavate all the members of the SRNF family, we searched the Phytozome v12.1 (see footnote 6) based on the conserved Short RING-like Zinc-Finger domain. We totally identified 140 *SRNF* genes in different dicotyledons (3 genes in *C*. *papaya*, 5 in *C*. *sativus*, 5 in *C*. *sativus*, 6 in *S*. *tuberosum*, 7 in *P*. *trichocarpa*, 8 in *M*. *truncatula*, 9 in *A*. *thailand*, 12 in *G*. *arboreum*, 8 in *G*. *raimondii*, and 24 in *G*. *hirsutum*), monocotyledons (3 in *A*. *trichopoda*, 6 in *O*. *sativa*, 7 in *Z*. *mays*, 8 in *H*. *vulgare*, and 10 in *S*. *bicolor*), moss (20 in *P*. *patens*), and fern (4 in *S*. *moellendorffii*), respectively. However, no *SRNF* gene was identified in picophytoplankton (*M*. *pusilla*) and algae (*Os*. *tauri, V*. *carteri*) (Figure 2B).

The rooted phylogenetic tree was constructed using full-length amino acid sequences, and the MEGA X software employed the neighbor-joining (NJ) method. Phylogenetic trees displayed consistent results including topologies of clusters and numbers as well as positions of genes in corresponding subfamilies. The largest subfamily SRNF-e with 54 members, 45 members in SRNF-a, 22 members in SRNF-b, 5 members in SRNF-c, 13 members in SRNF-d (Figure 2A). Interestingly, group SRNF-c and SRNF-d only exist in monocotyledons, while group SRNF-e only exists in dicotyledons. SRNF-a was the only group possessing *SRNF* genes from moss, fern, and several from monocotyledons, dicotyledons, suggesting that *SRNF* genes originated from moss and their evolution occurred before the separation of monocots and dicots. Also, *SRNFs* had suffered a great loss in the evolutionary progress from moss (*P*. *patens*) to fern (*S*. *moellendorffii*), which implies that plant-specific *SRNF* family genes were generated in the transition to a terrestrial environment. The results showed that no SRNF consensus was specific to cotton, as many species possess them, suggesting that the RING-like zinc-finger domain was highly conserved among plants.

### Protein Motifs and Cis-Acting Regulatory Elements Analysis

**Figure 3:**
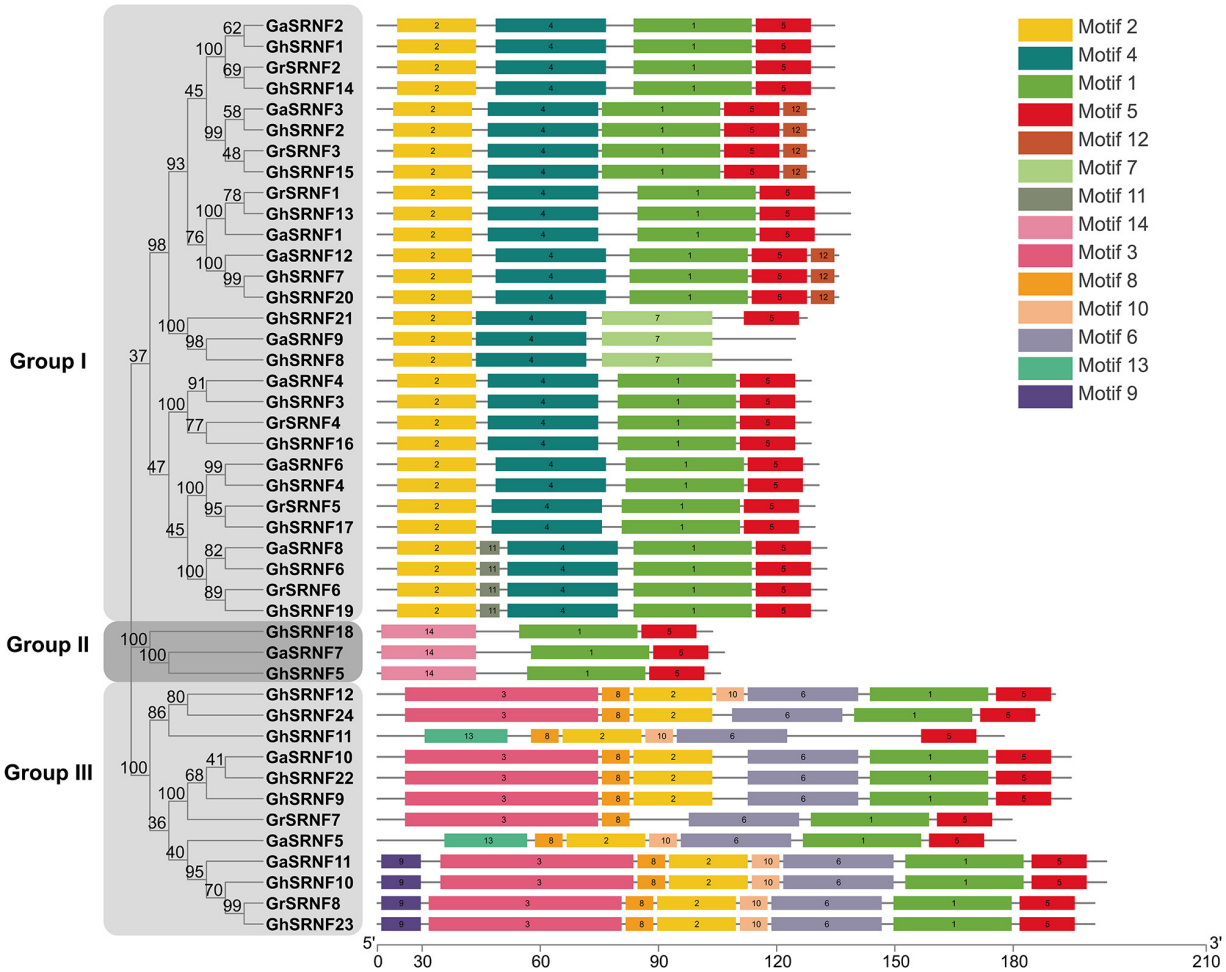
Protein level conserved motifs analysis. The conserved motifs of the SRNF proteins were identified by MEME (see footnote 8), and each motif was indicated by a numbered color box. The lengths of the motifs in each protein were drawn to scale.

To figure out the structure similarity of SRNF proteins in the same physical clusters, 14 putative motifs were characterized as motif 1 to motif 14 in the *GaSRNFs, GrSRNFs*, and *GhSRNFs*, and each protein had 3–8 motifs in their protein. The relative positions of the motifs in the same clade were found to have various patterns, *SRNF* genes with similar motif distribution patterns were clustered into three subgroups. Almost all the SRNFs contained motif 1 and motif 5, which meant they presented a typical SRNF domain. Respectively, Motif 1 was a combination of the RING domain and Zn_Tnp_IS1 domain (PF03811), and motif 5 was a Zn_Tnp_IS1 (PF03811) domain repeat, which located in the C-terminal (Figure 3). Further, Group II is an EAR motif-loss type, which may lead to GaSRNF7, GhSRNF5, and GhSRNF18 cannot act as TPL adaptors. TPL (TOPLESS) emerged as a corepressor to mediate transcriptional repression by interacting with a diverse range of EAR-motif contained transcription factors (Plant et al., 2021). Although other motifs have not been reported in plants, they might undertake important functions within organisms. Collectively, from 44 *SRNF* genes above, all members have one single exon throughout their open reading frames.

**Figure 4:**
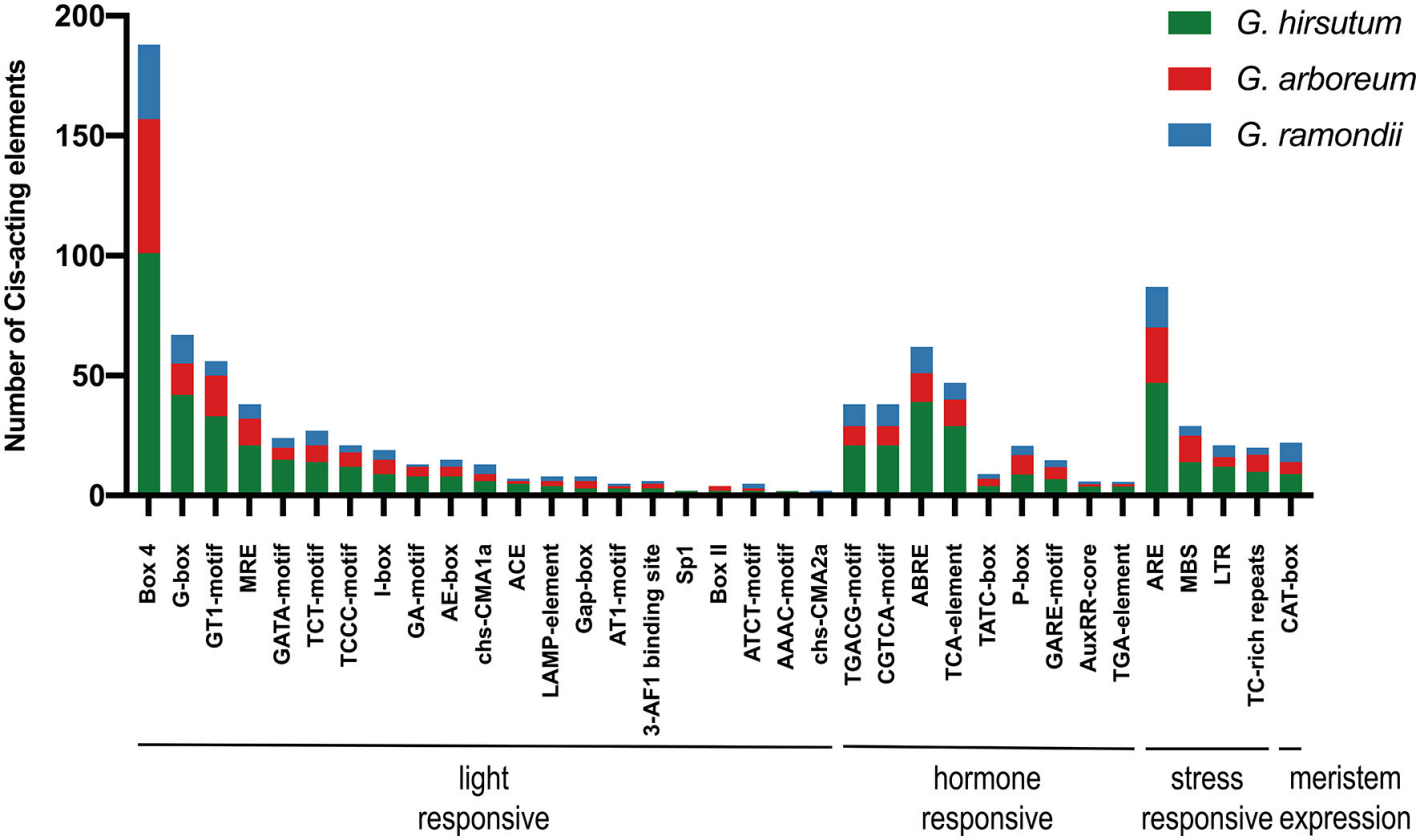
The 2 kb promoters of *SRNF* genes in *G. hirsutum, G. arboreum*, and *G. raimondii* were extracted and identified on PlantCARE (see footnote 9) online service. The statistical analysis of cis-acting regulatory elements was generated with element names and function types, different color refers to three cotton species.

As important transcription factor binding sites, cis-acting regulatory elements in the promoter region could provide information to the regulation of genes during plant development stages and their responses to various hormones and stresses. The 2 kb sequences upstream promoter regions were extracted and deployed on PlantCARE (see footnote 9) for potential cis-acting regulatory elements. Various cis-acting elements of *SRNF* genes from upstream were predicted to be highly related to light, phytohormones, defense, and stress responses. Most of the cis-acting elements were light-responsive, containing 188 for Box4, 67 for G-box, 56 for GT1-motif, 38 for MRE, and so on. The cis-acting regulatory elements involved in phytohormone responses include MeJA-responsive elements (TGACG-motif, CGTCA-motif), the ABA-responsive element (ABRE), gibberellin-responsive elements (TCA-element, P-box, and GARE-motif), and auxin-responsive elements (AuxRR-core and TGA-element). In addition, many *SRNF* genes had defense and stress-responsive elements, such as anaerobic-responsive element (ARE), MYB binding site for drought inducibility-responsive (MBS), low temperature-responsive (LTR) (Figure 4). Notably, each *SRNF* gene had a multi-copy of light-responsive elements, ranging from 4 to 18 motifs, implying that the expression of *SRNF* genes was mainly induced by light signaling pathways.

### Chromosomal Location and Synteny Analysis of *GhSRNF* Genes

**Figure 5:**
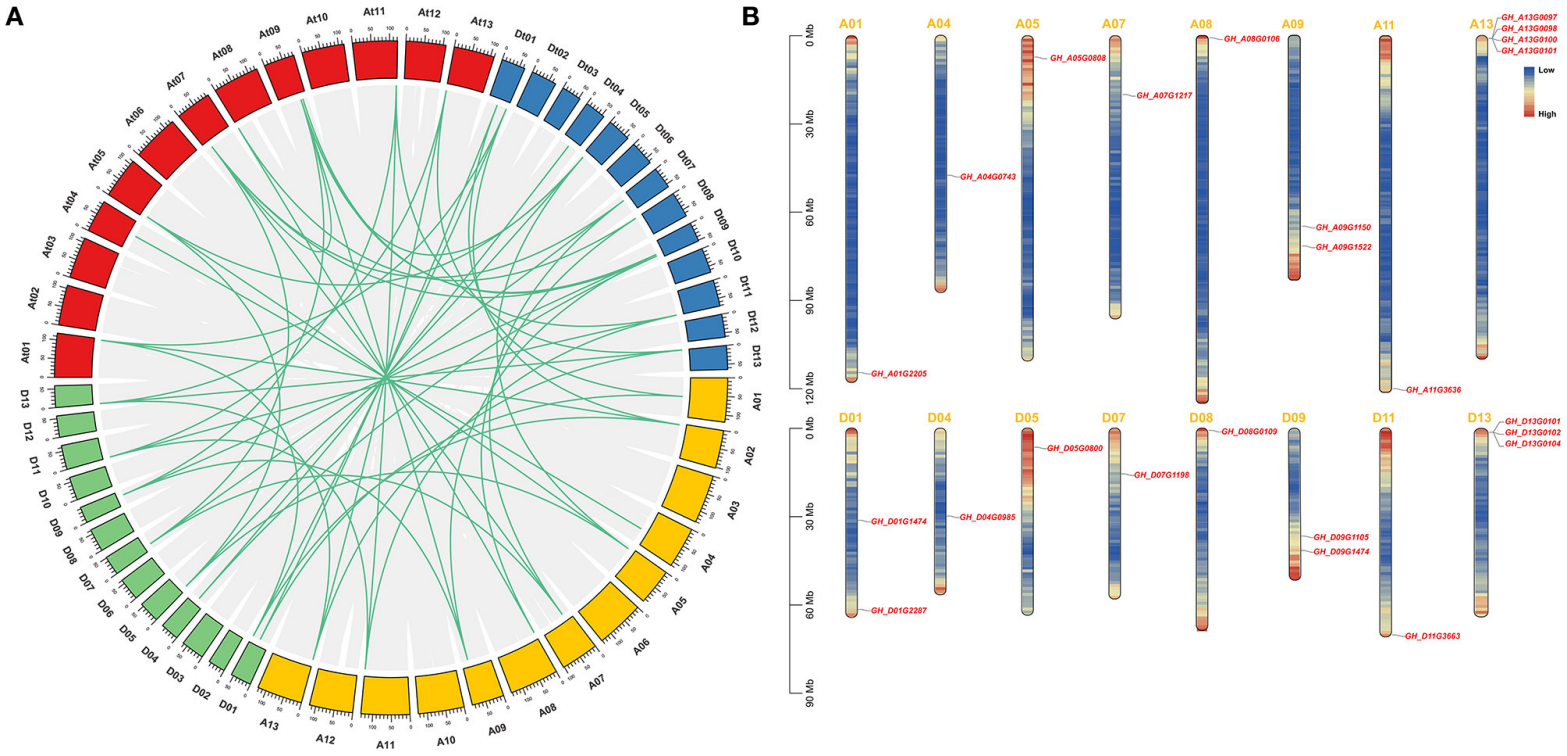
Collinear correlations between *G. hirsutum, G. arboreum*, and *G. raimondii*, and chromosomal location of the *GhSRNF* members. **(A)** Collinear correlations in *G. hirsutum, G. arboreum*, and *G. raimondii*. Gene duplication and collinearity analysis among *SRNF* genes in *G. hirsutum* (At and Dt subgenome), *G. arboreum* (A genome), and *G. raimondii* (D genome). Duplication gene pairs were linked by green lines. Scale bars at the top of the chromosomes are 10 Mb. **(B)** Genomic distributions of *GhSRNF* genes on *G. hirsutum* chromosomes. The *GhSRNFs* were plotted based on the physical location, length of chromosomes. Heatmap of each chromosome indicated the gene density by the frequency per 1 Mb.

According to the available *G*. *hirsutum* genome annotation, 24 identified *GhSRNF* genes were mapped onto corresponding chromosomes. Respectively, 11 *GhSRNF* genes were located on At subgenome chromosomes while 13 were on Dt subgenome chromosomes (Figure 5B). Some *GhSRNFs* made physical clusters with members of the same group at specific positions. For example, many *GhSRNF* genes in group SRNF-b located on At13 and Dt13, suggesting that the formation of these physical clusters may be due to tandem duplications. However, unlike *GaSRNF* and *GrSRNF*, there were no *GhSRNF* genes located on At02, Dt02, Dt05 chromosomes. The absence of *GhSRNF* genes on these chromosomes or uneven distributions illustrated translocation during evolution.

To investigate the expansion of *SRNF* genes, the synteny and collinearity analysis was performed by MCScanX between *G*. *arboreum, G*. *raimondii*, and *G*. *hirsutum*. Based on syntenic relationships, there are 12 paralogous gene pairs identified because of segmental duplications. Whole-genome duplication (WGD) resulted in 43 orthologous gene pairs, and of these, there were 9 pairs between the At subgenome and A genome, 7 pairs between the At subgenome and D genome, 8 pairs between the Dt subgenome and A genome, 11 pairs between the Dt subgenome and D genome, 7 pairs between orthologous chromosomes, and 1 orthologous gene pair between non-orthologous chromosomes (Figure 5A).

Furthermore, we calculated the non-synonymous (Ka) and synonymous (Ks) values, which showed that 39 duplicated pairs had Ka/Ks < 1.0, and among them, 28 duplicated pairs exhibited Ka/Ks values < 0.5. However, 6 duplicated gene pairs showed Ka/Ks values > 1.0 Collectively, cotton *SRNF* duplicated genes experienced positive selection pressures as a high proportion of Ka/Ks values of duplicated gene pairs were < 1.0.

### Expression Patterns of *GhSRNF* Family Genes

Previous reports had demonstrated that the *GIR*/*SRNF* genes play vital roles in root hair development and fiber elongation (Wu and Citovsky, 2017a,b; Feng et al., 2019; Wang et al., 2021). To identify the functions of *GhSRNF* genes at different developmental stages, the expression levels of *GhSRNFs* were evaluated from the published transcriptomes from CottonOmics (see footnote 10) during vegetative, reproductive growth stages, and fiber development stages. There were many great works done for four classic stages of initiation, elongation, secondary cell wall deposition (SCW), and maturation in fiber development (Wang et al., 2019).

**Figure 6:**
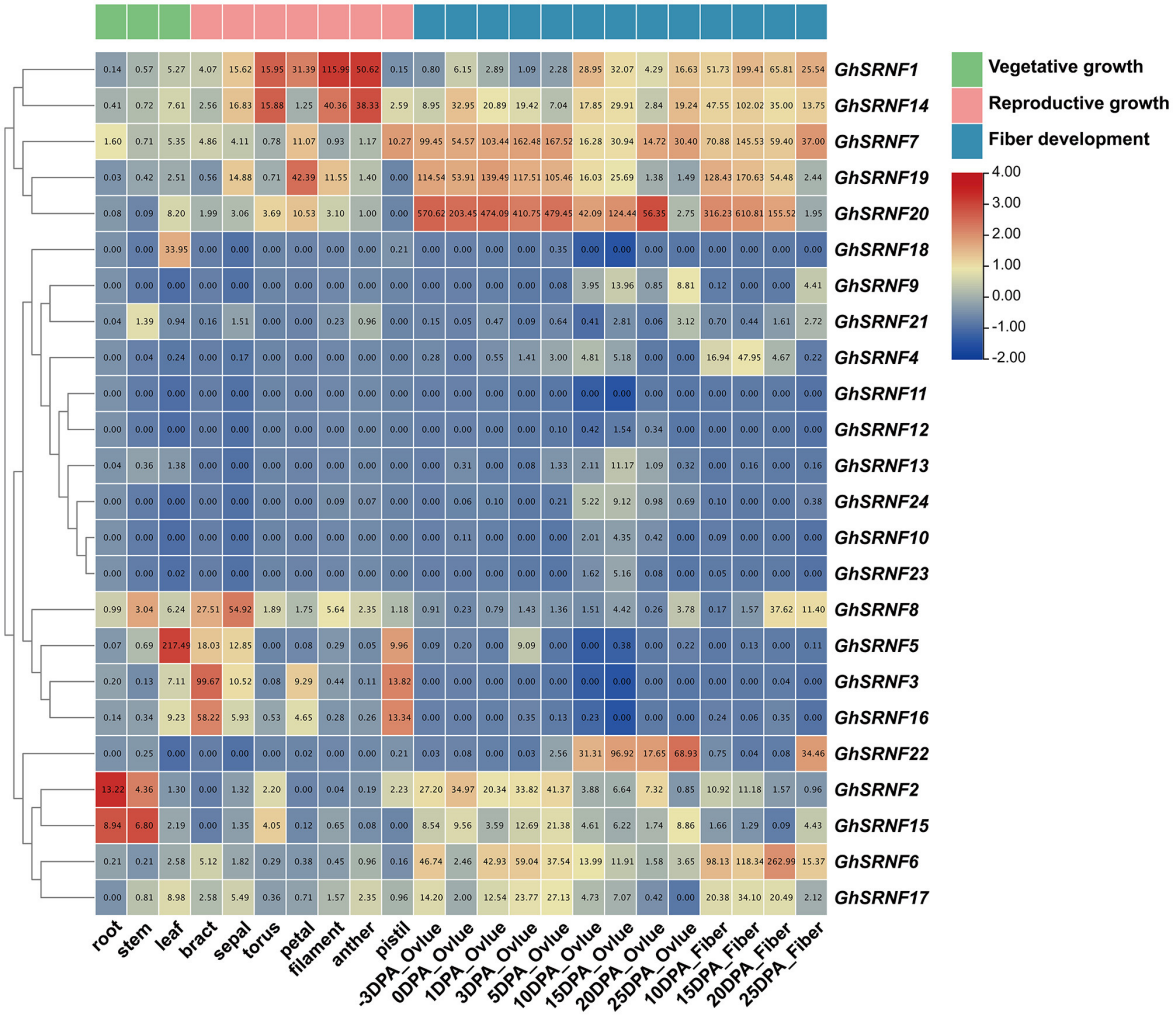
Temporal specificity expression analysis at different developmental stages of 24 *GhSRNF* genes in *Gossypium hirsutum acc*. *TM*-*1*. Expression patterns of *GhSRNF* genes from vegetative to reproductive growth stage, also fiber development stages in ovule and fiber were concluded. Heatmap was normalized by the average log_2_ of FPKM values. The cluster tree of *GhSRNF* genes shown on the left was based on the expression level.

To explore the expression patterns of *GhSRNFs* in *G*. *hirsutum acc*. *TM*-*1*, we extracted expression levels from published data of 12 different tissues (root, stem, leaf, bract, sepal, torus, filament, anther, pistil, ovule, fiber) at different developmental stages. As a result, genes depicting similar expression patterns were closely clustered. Many putative paralogous genes, such as *GhSRNF1* (At01) and *GhSRNF14* (Dt01), *GhSRNF7* (At09), and *GhSRNF19* (Dt09) showed similar expression patterns, suggesting redundant roles of these *GhSRNF* gene pairs. For example, *GhSRNF2*/*15* displayed relatively high expression levels in root and stem than that in leaf. At the reproductive stage, the expression levels of *GhSRNF1*/*14* were higher in stamen (filament and anther) than that in bract. In contrast, *GhSRNF3*/*16* were highly expressed in bract than that in other floral organs. At the fiber development stage, *GhSRNF7*/*20* were highly and constantly expressed in ovule at the initiation stage, *GhSRNF20* expression peaked at 3 DPA when expression levels were ~6-fold higher than at −3 DPA. In addition, *GhSRNF1*/*6*/*7*/*14*/*17*/*19*/20 increased from 10 DPA to 15 DPA in the fiber elongation stage, then decreased in the SCW deposition and maturation stages. However, *GhSRNFs* located on At13 and Dt13 exhibited unexpressed or expressed at a low level in most tissues above (Figure 6). Taking fiber development into consideration, the results suggested that *GhSRNF* genes displayed diverse spatiotemporal expression patterns at three developmental stages in *G*. *hirsutum*.

### Expression of *GhSRNF18* Positively Correlated With the PR Length

The *GhSRNF5/18* were characterized as EAR motif loss SRNF members and belonged to the subgroup II subfamily which consists of only two, one, and zero members in *G. hirsutum, G. arboreum*, and *G. raimondii*, respectively (Figure 3). In *Arabidopsis*, two EAR motifs-containing SRNF proteins GIR1/2 are involved in root hair development (Wu and Citovsky, 2017a). Cotton is cross-pollinated, and there were many genetic variations that existed among different cotton cultivars. Although gene sequences of *GaFZ* and its homologous gene of *GhSRNF5/18* are highly conserved, there are divergences in their promoter regions (Feng et al., 2019; Wang et al., 2021), suggesting that their expression patterns may vary greatly among different cotton cultivars. Consequently, GhSRNF18 probably are involved in root development in some upland cotton cultivars, although the level of *GhSRNF18* expression in roots of *TM-1* was relatively low (Figure 6).

**Figure 7:**
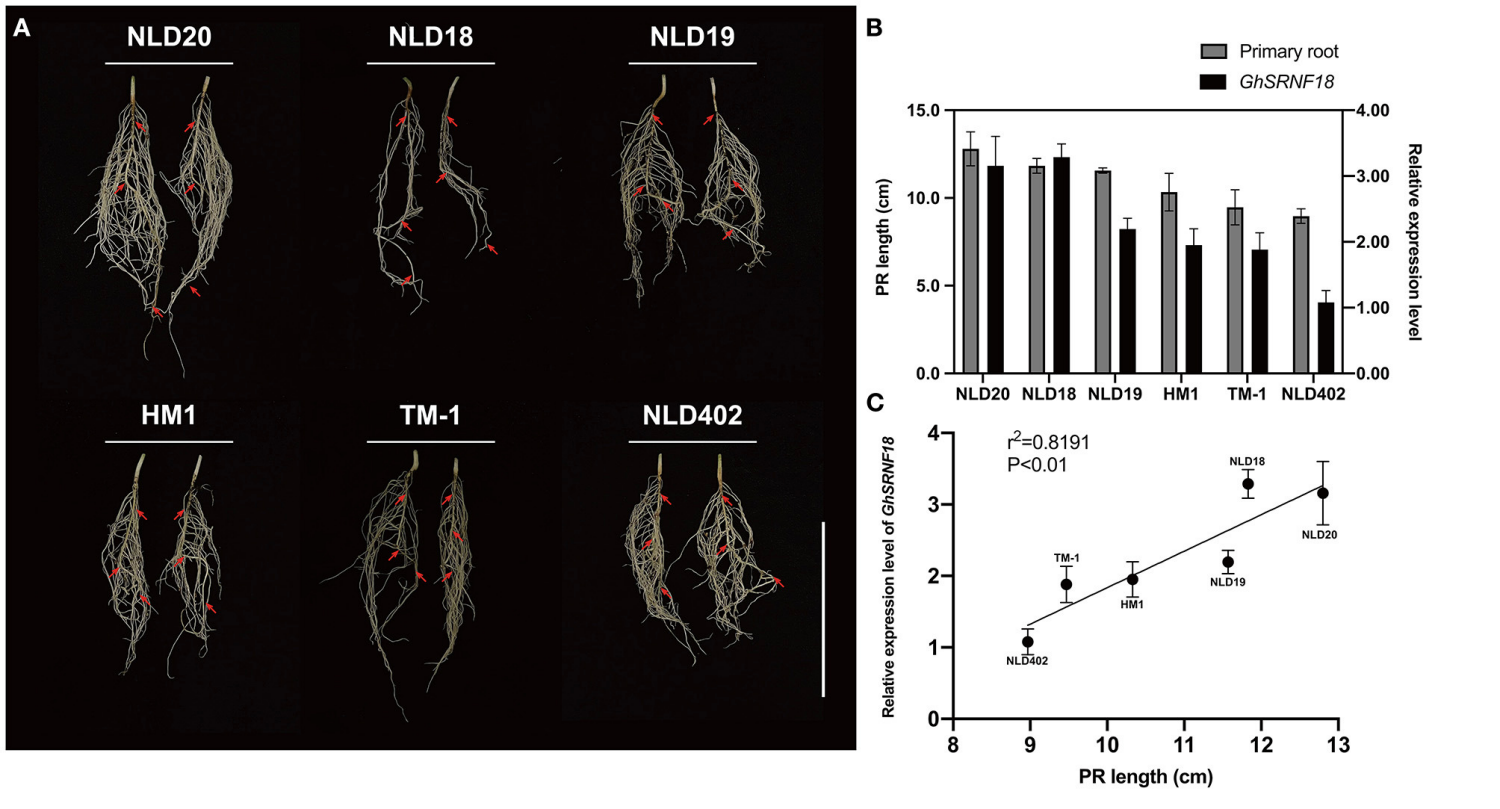
The expression pattern of *GhSRNF18* positively correlated with primary length (PR) in six *G. hirsutum* accessions. **(A)** The root system phenotype of six *G. hirsutum* accessions. Red arrows represent the PR. Scale bar is 5 cm. **(B)** The PR length and the expression level of *GhSRNF18* in six *G. hirsutum* accessions, the error bars represent the means ± SEM. **(C)** The Pearson correlation analysis between the relative expression value and the PR length (means ± SEM; *P* < 0.01; *R* squared = 0.8191, Pearson correlation).

To determine whether *GhSRNF5*/*18* genes were involved in root system regulation, their root expression level in primary roots was quantified and compared among six *G*. *hirsutum* accessions. First, we observed the root phenotypes in six cultivars. Among them, *NLD19*/*20* had advantages in the primary root (PR) length with abundant lateral roots (LRs) (Figure 7A). The PR length was higher in the *NLD18*/*19*/*20* cultivars than *HM1*/*TM*-*1*/*NLD402*. Similarly, *GhSRNF18* was highly expressed in the longer PR length cultivars as described (Figure 7B). However, even though *GhSRNF5* was expressed higher in roots, there was no correlation with PR length. Furthermore, we performed a Pearson correlation analysis, which suggested that the expression level of *GhSRNF18* was positively correlated with PR development, especially in *NLD20*/*402* and *HM1* (Figure 7C).

### Ectopic Expression of *GhSRNF18* Regulates the PR Growth

**Figure 8:**
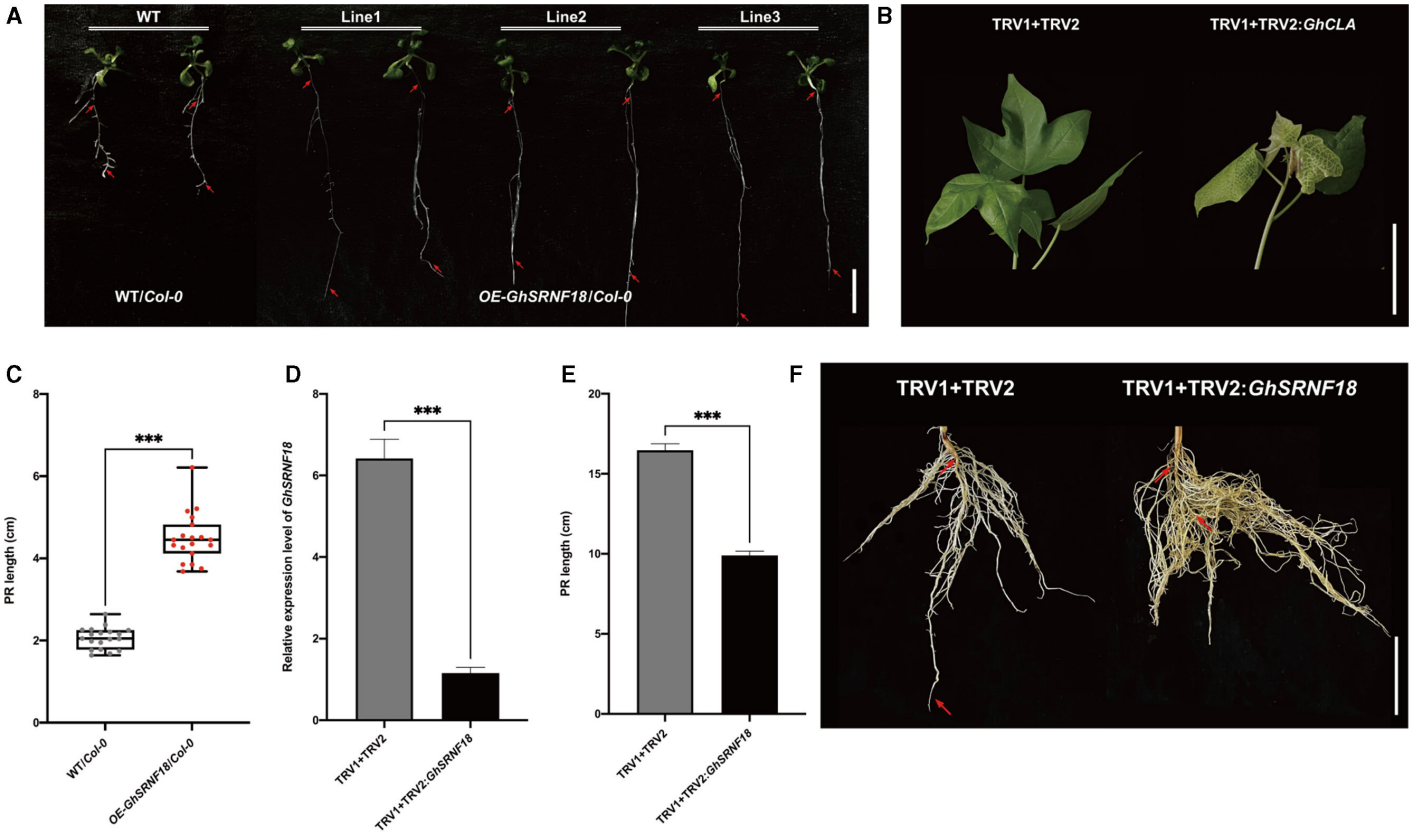
*GhSRNF18* in *Arabidopsis* enhanced primary root (PR) growth **(A)** Phenotype of three independent *OE-GhSRNF18* transgenic lines reveals GhSRNF18 promotes PR length. Red arrows represent the primary root (PR), scale bar is 1 mm. **(B)** The albino phenotypes of TRV1 + TRV2: *GhCLA* (positive control) plants. **(C)** The PR length comparison between WT/*Col-0, OE-GhSRNF18*/*Col-0* seedlings grown for 14 d on 1/2 MS medium (means ± SEM, *n* = 20; ^***^P < 0.001; one-way ANOVA test) **(D)** The PR length and Relative expression level comparison between controls: TRV1 + TRV2 and TRV1 + TRV2:*GhSRNF18* (means ± SEM; ^***^P < 0.001; student's *t*-test) **(E)** Phenotypes of TRV1+TRV2 (negative control) and TRV1 + TRV2:*GhSRNF18* plants. Scale bars are 5 cm in **(B,F)**.

To determine the role of GhSRNF18 in the PR growth, three transgenic *Arabidopsis* overexpression lines of *GhSRNF18* driven by the constitutive 35S promoter was generated. Although the amino acid sequences of GhSRNF18 were best matched to GIR2 in *Arabidopsis*, GhSRNF18 was an EAR-motif loss type as compared with the AtGIR2 sequence. Thus, compared with WT/*Col-0* plants, the transgenic plants overexpressing *GhSRNF18* showed significant elongation in PR length with relatively low emergence of lateral roots. The statistical data indicated that the PR length of *OE-GhSRNF18* lines increased to 217.9% as compared with WT/*Col*-*0* plants (Figures 8A,C). These results implied the ectopic expression of *GhSRNF18* regulates the PR growth in *Arabidopsis*.

To further explore the function of *GhSRNF18* in cotton PR growth, *GhSRNF18* was silenced using VIGS (virus-induced gene silencing) system in *NLD20*. Until the albino phenotype appearance in the leaf, we monitored the relative expression level of the control lines (TRV1 + TRV2) and VIGS line (TRV1 + TRV2:*GhSRNF18*) using qRT-PCR to confirm the silenced expression of *GhSRNF18* in the VIGS line (Figure 8B). The results showed that TRV2:*GhSRNF18* plants had hindered the PR growth, while the PR length of the TRV2:*GhSRNF18* was significantly suppressed 39.8% as compared with the control lines (Figure 8D). All these results above strengthened our hypothesis that the *GhSRNF18* promotes PR growth.

### GhSRNF18 Integrates Multiple Signaling Pathways to Promote PR Growth

**Figure 9:**
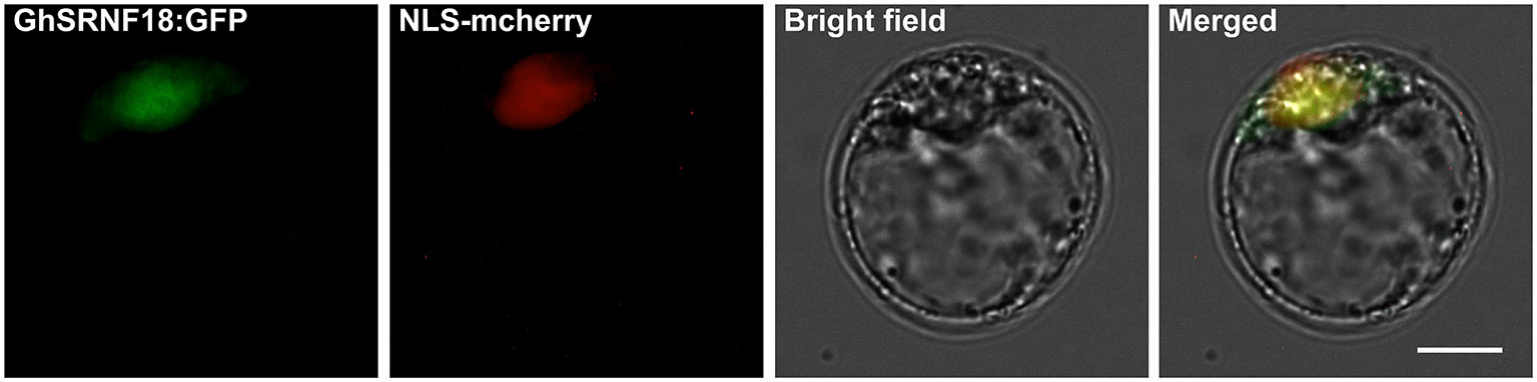
Subcellular localization of the GhSRNF18 protein in *Arabidopsis* protoplast. GhSRNF18:GFP (GhSRNF18 and green fluorescent protein fusion protein) and NLS-mcherry (nuclear localization sequence-fused mCherry protein) were co-transformed into protoplast. The scale bar is 10 μm.

In *Arabidopsis*, GIR1 and GIR2 interact with GL2 and TPL proteins in the nucleus due to the BiFC assays (Wu and Citovsky, 2017a,b). GhSRNF18 orthologous GaSRNF7 (GaFZ) protein was observed predominantly in the membrane and nucleus (Wang et al., 2021). The results of subcellular localization assays in *Arabidopsis* protoplast showed GhSRNF18 distributed in the nucleus (Figure 9), which implies that GhSRNF18 may control the expression level of several PR-related genes.

**Figure 10:**
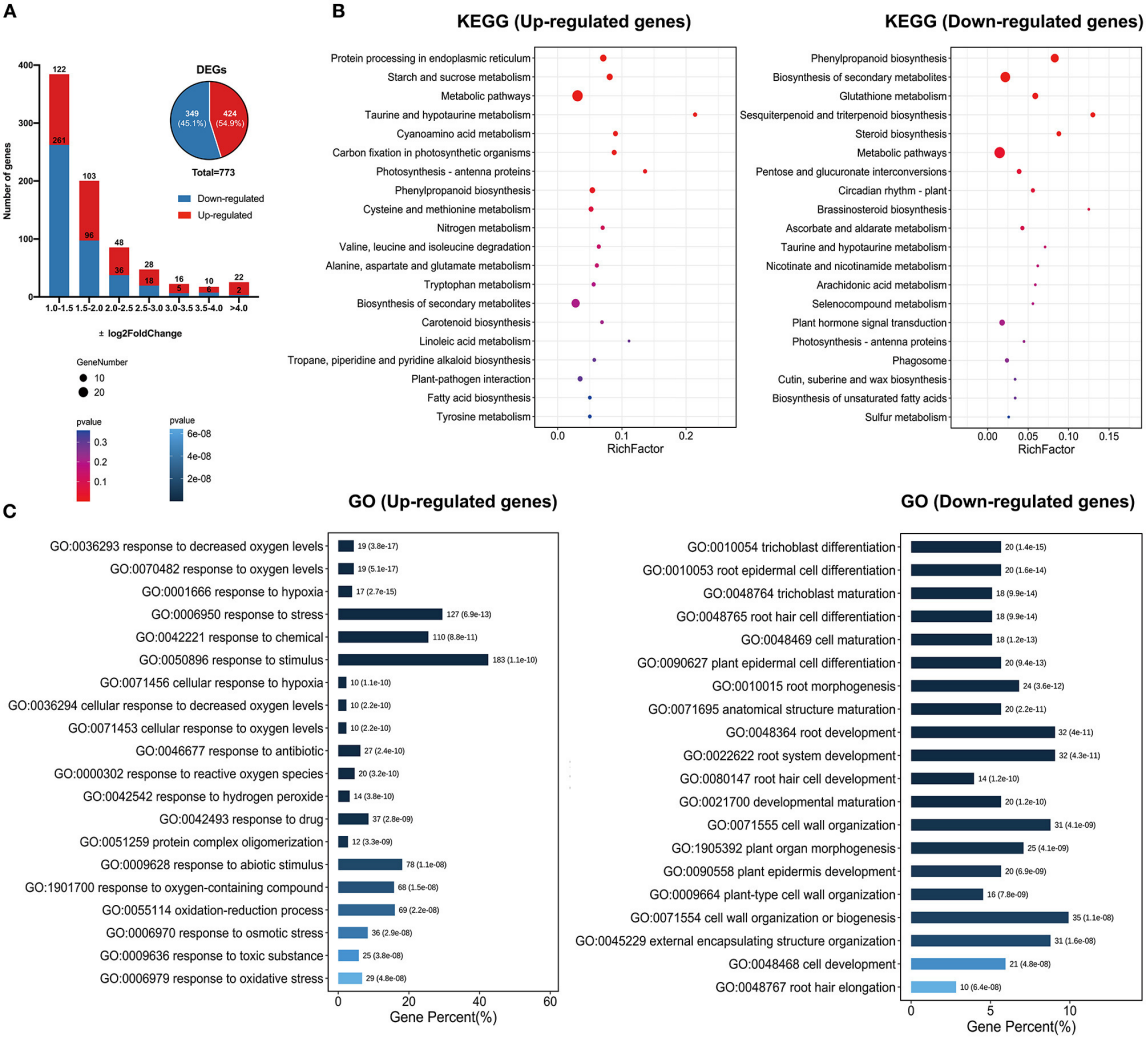
KEGG and GO enrichment results of non-redundant DEGs between *OE-GhSRNF18* lines and *Col-0* in Arabidopsis. **(A)** Differentially expressed genes number. between *OE-GhSRNF18* and *Col-0*. Up-regulated and Down-regulated genes distribution are based on log_2_Fold Change. **(B)** KEGG pathway analysis of up-regulated and down-regulated genes. Enrichment scattered bar plots are based on the gene number and *p*-value. **(C)** GO terms enrichment analysis of up- and down-regulated genes. The length of the bars indicated the gene percent, the *p*-value is displayed by the blue gradient.

To explore the mechanisms of *GhSRNF18* genes in regulating PR growth and root system development, we identified the differentially expressed genes (DEGs) between *OE*-*GhSRNF18* lines and WT/*Col*-*0*. Among 27416 expressed genes, only ~783 genes were differentially expressed, comprising ~424 up-regulated genes (54.9% of all non-redundant DEGs) and ~359 down-regulated genes (54.9% of all non-redundant DEGs) in *OE*-*SRNF18* lines (Figure 10A).

Then, we analyzed the KEGG pathway of up-regulated and down-regulated genes separately, indicating that genes for protein processing in the endoplasmic reticulum, starch and sucrose metabolism, metabolic pathway, taurine and hypotaurine, cyanoamino acid metabolism were upregulated. In contrast, genes related to phenylpropanoid biosynthesis, circadian rhythm, biosynthesis of secondary metabolites, glutathione metabolism, sesquiterpenoid, and triterpenoid biosynthesis were downregulated (Figure 10B).

Furthermore, GO terms for up-regulated genes were significantly enriched in hypoxia response as well as in abiotic stress stimulus-response. However, down-regulated genes were found to be enriched in root hair differentiation and trichoblast maturation (Figure 10C). These data suggested that the ectopic expressions of *GhSRNF18* inhibited differentiation gene expression and promote starch and sucrose-related gene expressions, which might be essential for PR growth.

**Figure 11:**
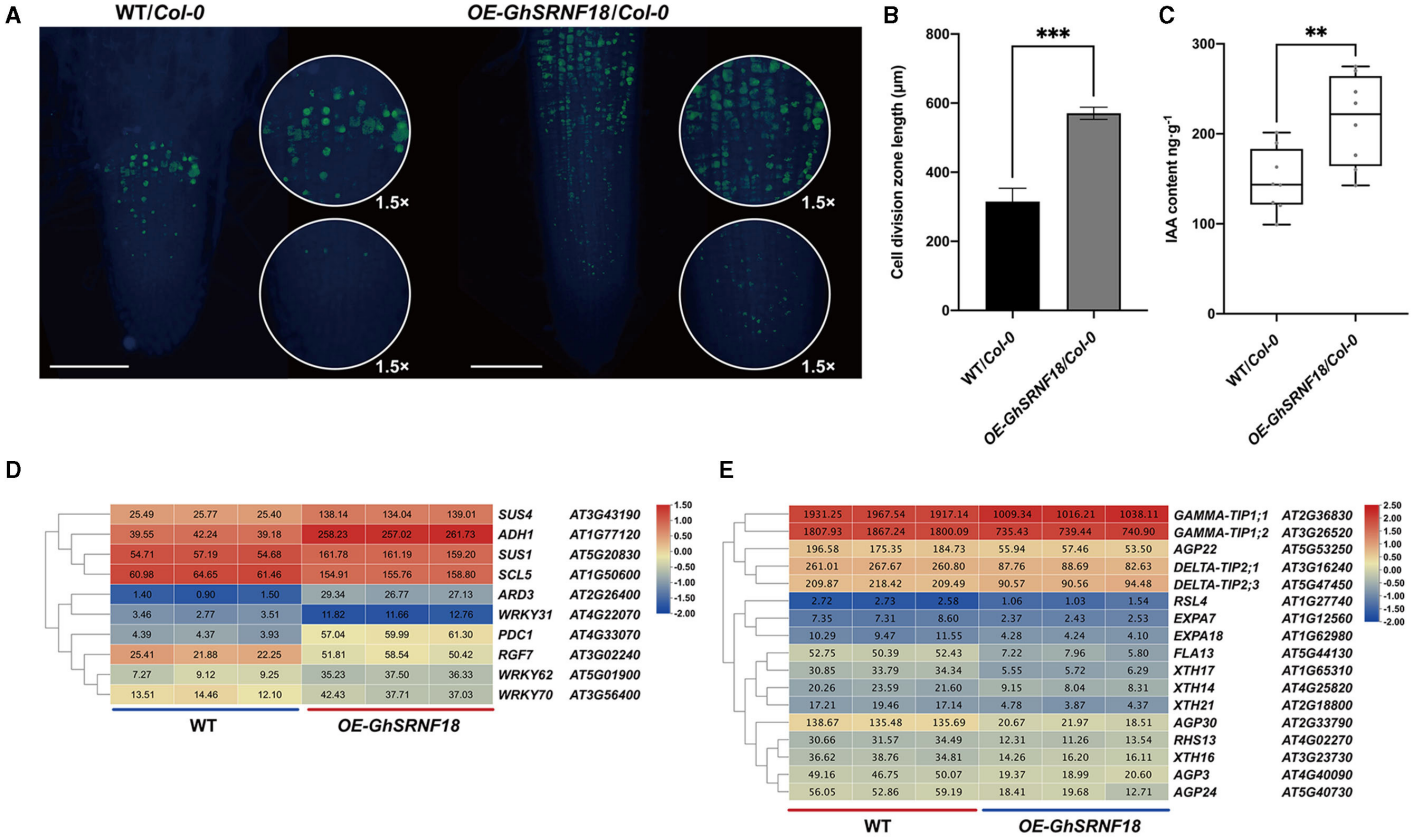
The downstream of GhSRNF18 regulate the auxin pathway and affect RAM activity. **(A)** EdU signals were detected with Alexa Fluro 488 azide (green panel). 1.5 times view is in the circle. Scale bars are 500 μM. **(B)** Length of cell division zone (means ± SEM; *n* = 3, ^***^P < 0.001 student's *t*-test). **(C)** IAA contents comparison between WT/*Col-0* and *OE-GhSRNF18*/*Col-0*. Quantitation of 8 biological replicates of the experiment is shown (means ± SEM; ^**^P < 0.05 student's *t*-test). **(D)** Heatmap of upregulated genes. **(E)** Heatmap of down-regulated genes. Heatmap was normalized by the average of log_2_ FPKM values. The cluster tree of *GhSRNF* genes shown on the left was based on the expression level.

Based on the *Arabidopsis* genomics associated with the Plant Ontology annotations, *SUS1*/*4* (SUCROSE SYNTHASE), *SCL5* (SCARECROW-LIKE), *WRKY31*/*62*/*70* (WRKY DNA-BINDING PROTEIN), *RGF7* (ROOT MERISTEM GROWTH FACTOR), *ARD3* (ACIREDUCTONE DIOXYGENASE), *ADH1* (ALCOHOL DEHYDROGENASE) were up-regulated in *OE*-*GhSRNF18*. However, *FLA13* (FASCICLIN-LIKE ARABINOGALACTAN PROTEIN), *AGP3*/*22*/*24* (ARABINOGALACTAN PROTEIN), *GAMMA*-*TIP1;1*/*1;2* (GAMMA INTRINSIC PROTEIN 1), *DELTA-TIP2;1*/*2;3* (DELTA TONOPLAST INTEGRAL PROTEIN 2), *XTH14*/*16*/*17*/*33* (XYLOGLUCAN ENDOTRANSGLUCOSYLASE/HYDROLASE), *RSL4* (ROOT HAIR DEFECTIVE 6-LIKE), *RHS13* (ROOT HAIR SPECIFIC), *EXPA7*/*18* (EXPANSIN) were down-regulated (Figures 11D,E). The GRAS transcription factor SCR is required to establish the QC identity and maintenance of root stem cell activity (Sabatini et al., 2003; Petricka et al., 2012). Among them, RGF/CLEL/GLV peptide signaling peptides modulate accumulation of auxin in root tips probably by regulation of *PLT1* and *PLT2* expressions to maintain root stem cell activities (Petricka et al., 2012; Whitford et al., 2012; Ou et al., 2016; Motte et al., 2019). Thus, the mass fraction of each endogenous IAA in the *OE*-*GhSRNF18*/*Col*-*0* and WT/*Col*-*0* root tissues was displayed by enzyme-linked immunosorbent assays. An ~1.4-fold increase was observed for the IAA mass fractions in *OE*-*GhSRNF18*/*Col*-*0* lines compared with WT (Figure 11C). Consistent with this, the EdU-labeled cell division zone in *OE-GhSRNF18* roots was ~1.8-fold larger than WT plants, suggesting that the RAM activity was increased in *GhSRNF18* overexpression lines (Figures 11A,B).

## Discussion

The root is one of the main vegetative organs of plants, which is responsible for water and minerals absorption, for the anchorage of plants in the soil, and for pathogen defense (Schachtman and Goodger, 2008; Petricka et al., 2012; Rich-Griffin et al., 2020). Primary roots growth is determined by the rate of cell division in the RAM and cell elongation in the EZ, which is tightly regulated by a series of transcription factors and multiple phytohormones (Petricka et al., 2012; Vanstraelen and Benkova, 2012; Motte et al., 2019). Adaptor proteins GIR1 and GIR2 have proved to interact with a negative transcriptional regulator of root hair formation GL2 and an epigenetic regulator TPL in *Arabidopsis*, affecting the root hair development (Wu and Citovsky, 2017a,b). In this study, 44 GIR-like proteins were identified in *G*. *arboreum, G*. *raimondii*, and *G*. *hirsutum* by sequence alignments using the GIR1 and GIR2 protein sequences (Supplementary Table 2). As all proteins above contain a conserved SRNF domain (CX_2_CX_12_CX_2_X_10_) (Figures 1B,C), we rename the GIR proteins as the SRNF family proteins. Some studies have considered that Zinc-finger proteins as transcription factors serving a wide variety of biological functions by binding DNA, RNA, proteins, or small molecules (Laity et al., 2001). Our results indicated that most SRNF members in *G*. *hirsutum* were predicted to be localized in the nucleus while others were predicted to be localized in the chloroplast (Supplementary Table 2). Nucleus, chloroplast, and mitochondria contain a genome that needs to be maintained, expressed, replicated, and repaired in a strictly controlled manner (Blomme et al., 2017). Therefore, the SRNF members in *G*. *hirsutum* tend to be potential regulatory factors for the expression of nucleus and chloroplast genes. We further identified 144 *SRNF* family genes in *M*. *pusilla, Os*. *tauri, V*. *carteri, P*. *patens, A*. *trichopoda, C*. *papaya, S*. *moellemdorffii, C*. *sativus, S*. *tuberosum, O*. *sativa, P*. *trichocarpa, Z*. *mays, H*. *vulgare, M*. *truncatula, A*. *thaliana, S*. *bicolor, G*. *arboreum, G*. *raimondii*, and *G*. *hirsutum*. The results showed the 144 *SRNF* genes above were divided into 5 subfamilies (SRNF-a to SRNF-e). Interestingly, the *SRNF* family genes appear to exist in lower plants (*P*. *patens*) and flowering plants but not in algae (*M*. *pusilla, Os*. *tauri, V*. *carteri*), which indicates that the moss may have acquired the *SRNF* family genes during the evolutionary transition of plants from aquatic to terrestrial environments. In addition, the numbers of *SRNF* family genes had increased as genome size decreased except *P*. *patens* (Figure 2B). In *P*. *patens*, there are 20 *SRNF* family genes, all of which belong to a subfamily SRNF-a (Figures 2A,B). However, the numbers of *SRNF* family genes in *P*. *patens* are much more than those in the diploid flowering plant as we identified, revealing the potential *SRNF* gene loss that occurred in the evolution from moss to higher plants. Besides, we identified 24 *SRNFs* in *G*. *hirsutum*, 12 *SRNFs* in *G*. *arboreum*, and 8 in *G*. *raimondii* (Figure 2B). These results indicate that *SRNF* gene gain events occurred in *G*. *hirsutum*, which is not consistent with the higher rate of gene loss in allotetraploid cotton than in both diploid species (Li et al., 2015; Zhang et al., 2015).

To further figure out the structure similarity of SRNFs, we investigated their conserved protein motifs using the MEME (see footnote 8) software and found an interesting phenomenon that the SRNFs in *G*. *arboreum, G*. *raimondii*, and *G*. *hirsutum* were segregated into two classes namely, EAR motif (LXLXL) contained (all of the group I) or EAR motif loss (group II and part of group III) (Figures 1A, 3, and Supplementary Figure 1). The EAR motif characterized by the consensus sequence of LXLXL or DLNXXP is a principal transcriptional repression motif in plants (Causier et al., 2012a,b). Thus, we speculated that the SRNFs containing the EAR motif probably is a novel transcription repressor while other SRNFs exert transcriptional regulatory activity through the interaction with other transcription factors.

Jasmonic acid and abscisic acid (ABA) are key regulators of plant responses to biotic and abiotic stresses, which is responsible for defense against insects and pathogens and for adaption under drought and salinity stress, respectively (Wasternack and Hause, 2013; Kuromori et al., 2018). As described in the analysis of cis-acting elements in the promoter of GhSRNF genes, MeJA-responsive (TGACG-motif, CGTCA-motif), ABA-responsive [abscisic acid-responsive element (ABRE)], and stress-responsive [anaerobic-responsive element (ARE)], MYB binding site for drought inducibility-responsive (MBS), [low temperature-responsive (LTR)] were significantly enriched on their promoters (Figure 4), thus indicating the potential roles of *GhSRNFs* under biotic and abiotic stresses.

We investigated the relationship between *GhSRNF18* expression and PR length in several upland cotton cultivars and our results suggested that the PR length showed a positive correlation with GhSRNF18 expression level (Figures 7A–C). To further confirm the role of GhSRNF18 in the regulation of PR growth, *GhSRNF18* was isolated from *TM*-*1* and transformed into *Arabidopsis*. As a result, over-expression of *GhSRNF18* in *Arabidopsis* promoted the PR growth (Figures 8A,C) while silenced *GhSRNF18* expression in *NLD20* (*GhSRNF18* expression level was relatively high in the root of this cultivar) using the VIGS system resulted in shorter PRs (Figures 8B,E,F). In addition, the *GhSRNF18* was localized in nuclei (Figure 9), which was the same as predicted. To further figure out the possible mechanisms of *GhSRNF18* involved in the PR growth, transcriptome sequencings of the wild type (WT)/*Col-0* and *GhSRNF18* overexpression lines were displayed, and these data were used for DEGs analysis and gene annotations. Subsequently, the present study performed GO and KEGG pathway analysis for these DEGs. The results revealed that the expression level of several genes involved in root hair, such as RHD proteins and EXPA proteins, significantly decreased in *GhSRNF18* overexpression lines (Figure 11D). In *Arabidopsis*, RHD6, RHD2, EXPA7, and EXPA18 have been proved to be involved in root hair morphogenesis by molecular genetics studies (Grierson et al., 2014). Meanwhile, a ~1.87-fold root hair linear density decrease was observed in *GhSRNF18* over-expression lines, indicating another role of *GhSRNF18* overexpression lines in the regulation of root hair development (Supplementary Figures 2A,B). We further identified the upregulated genes related to PR growth in *GhSRNF18* overexpression lines. The results showed that *SCL5* and *RGF7* significantly increased in *GhSRNF18* overexpression lines comparing with the WT (Figure 11E). Our data indicated increased auxin content in *GhSRNF18* overexpression lines (Figure 11D), the RAM activity was increased in *GhSRNF18* overexpression lines as well (Figures 11A–C). Taken together, GhSRNF18 promoted PR growth via modulation of auxin accumulation in the root and RAM activity. A higher endogenous IAA level also hints that there might be more auxin transported from shoots to roots, thereby affecting root system architecture (PR and LRs).

## Data Availability Statement

The original contributions presented in the study are publicly available. This data can be found at: National Center for Biotechnology Information (NCBI) BioProject database under accession number PRJNA743223.

## Author Contributions

CS conceived and designed the research. LY, HL, YWa, YWe, and QZ performed the experiments. SZ and XR analyzed the data. CS and LY contributed to writing the manuscript. LY, CS, and JR modified and revised the manuscript. All authors read and approved the final manuscript.

## Funding

This research was sponsored by the Natural Science Foundation of Zhejiang Province, China (LQ19C020006), the National Key R&D Program for Crop Breeding (2016YFD0101417), the Program for Research and Development of the Zhejiang A&F University (2018FR038), and Zhejiang Xinmiao Talents Program Key project at central government level (2020R412002).

## Conflict of Interest

The authors declare that the research was conducted in the absence of any commercial or financial relationships that could be construed as a potential conflict of interest.

## Publisher's Note

All claims expressed in this article are solely those of the authors and do not necessarily represent those of their affiliated organizations, or those of the publisher, the editors and the reviewers. Any product that may be evaluated in this article, or claim that may be made by its manufacturer, is not guaranteed or endorsed by the publisher.

## Footnotes

^1^ https://www.arabidopsis.org/

^2^ https://www.cottongen.org/

^3^ https://smart.embl.de/

^4^ https://web.expasy.org/compute_pi/

^5^ http://www.csbio.sjtu.edu.cn/bioinf/plant/

^6^ https://phytozome.jgi.doe.gov/

^7^ https://www.evolgenius.info/evolview/

^8^ https://meme-suite.org/meme/

^9^ http://bioinformatics.psb.ugent.be/webtools/plantcare/html/

^10^ http://cotton.zju.edu.cn/

^11^ http://www.omicshare.com/tools
