# Supplementary material for: Genome-Wide Analysis of SRNF Genes in Gossypium hirsutum Reveals the Role of GhSRNF18 in Primary Root Growth
Source: Front Plant Sci. 2021 Sep 22;12:731834. doi: 10.3389/fpls.2021.731834 (PMC8494181; doi:10.3389/fpls.2021.731834)
Supplement: Supplementary file 2 [file Data_Sheet_2.docx]

Supplementary Material

## Supplementary Figure


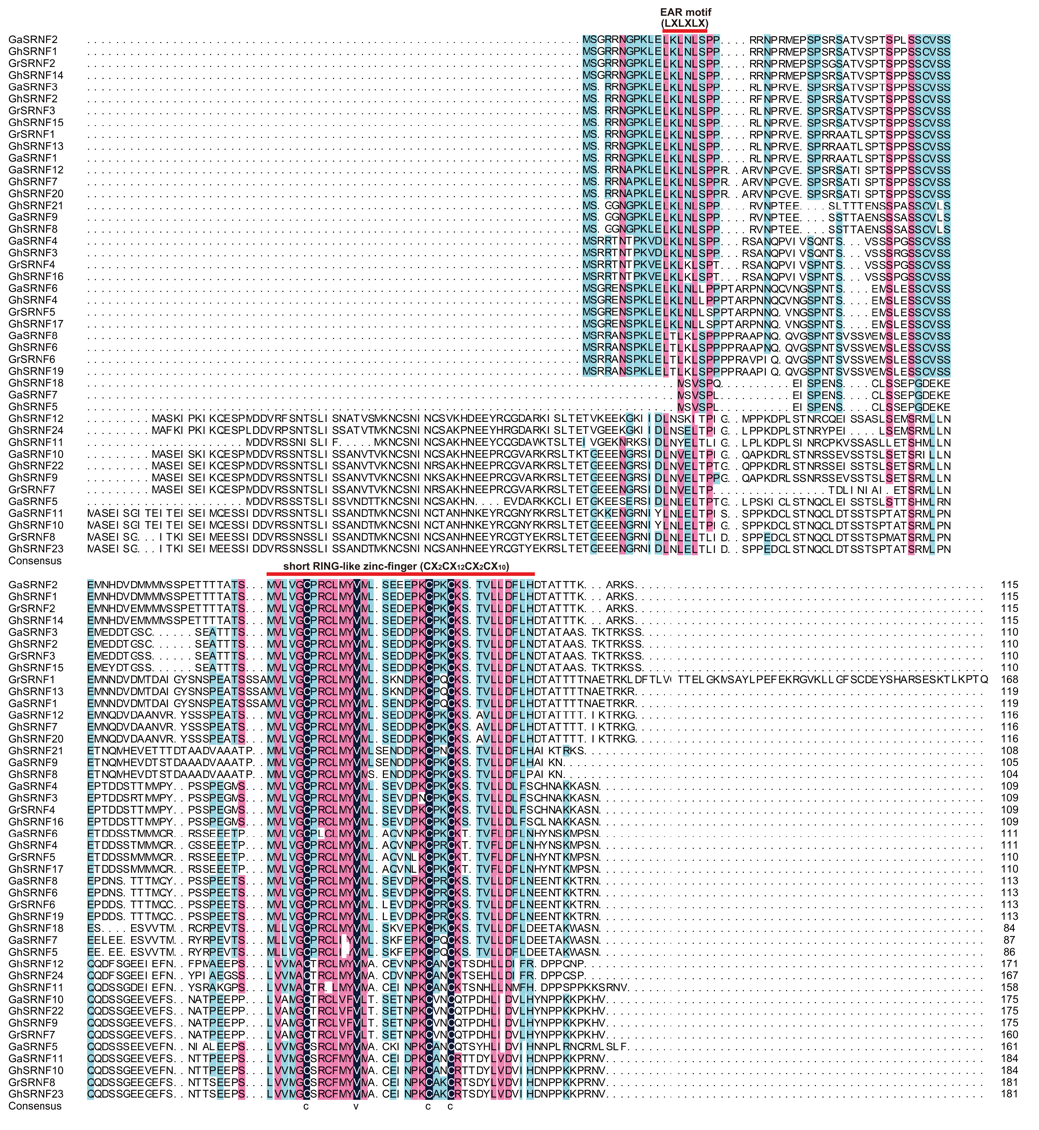


**Supplementary Figure 1.** Multiple alignment of protein sequences of the SRNF family in *G*. *arboreum*, *G. raimondii*, *G*. *hirsutum*. Colour code as follws: black sequence, 100%; pink sequence, ≥75%; blue sequence, ≥50%.

**
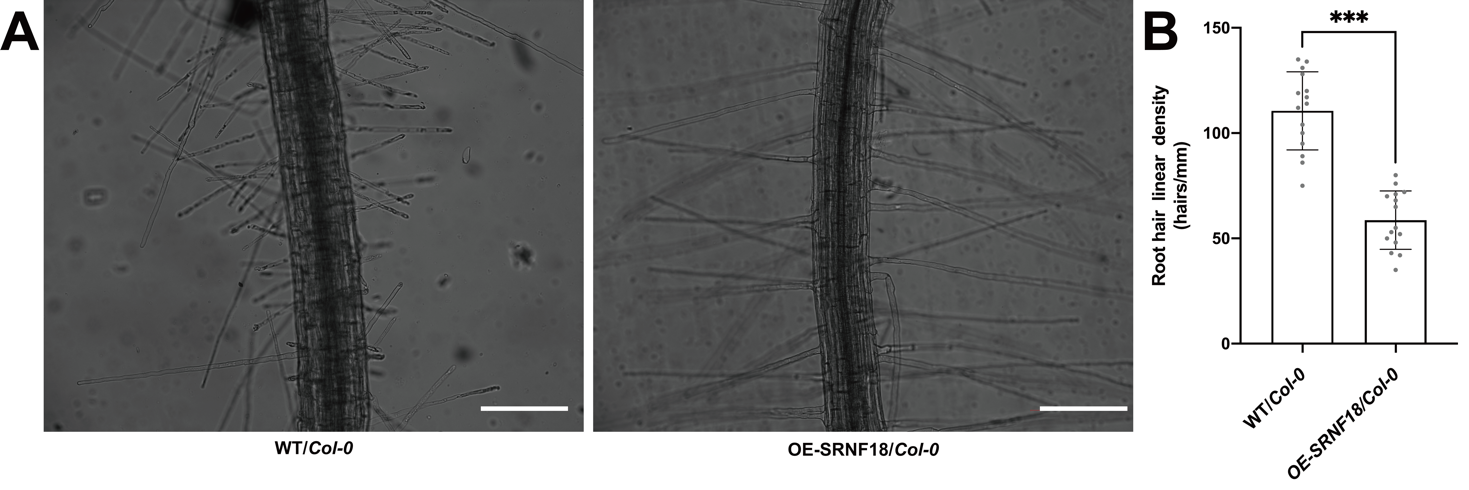
**

**Supplementary Figure 2.** Effects of *GhSRNF18* on root hair development. (A) Root hair density comparison between 14 d seedlings of *GhSRNF18* overexpression lines and WT plants in *Arabidopsis*. Scale bars are 200μM. (B) Root hair linear density comparison of 14 d seedling between WT/*Col-0* and transgeneic line *OE-SRNF18*/*Col-0*.
